# The Role of Transmission Electron Microscopy in the Early Development of Mesoporous Materials for Tissue Regeneration and Drug Delivery Applications

**DOI:** 10.3390/pharmaceutics13122200

**Published:** 2021-12-20

**Authors:** María Luisa Ruiz-González, Almudena Torres-Pardo, José M. González-Calbet

**Affiliations:** 1 Departamento de Química Inorgánica, Facultad de Químicas, Universidad Complutense de Madrid, 28040 Madrid, Spain; luisarg@ucm.es (M.L.R.-G.); atorresp@ucm.es (A.T.-P.); 2 ICTS ELECMI Centro Nacional de Microscopia Electrónica, Universidad Complutense de Madrid, 28040 Madrid, Spain

**Keywords:** mesoporous materials, nanomaterials, drug delivery, tissue regeneration, transmission electron microscopy, energy dispersive spectroscopy, STEM-EELS

## Abstract

For the last 20 years, silica-based mesoporous materials have provided a sound platform for the development of biomedical technology applied to tissue engineering and drug delivery. Their unique structural and textural characteristics, chiefly, the ordered distribution of homogeneous and tunable pores with high surface areas and large pore volume, and their excellent biocompatibility provide an excellent starting point for bone tissue regeneration on the mesoporous surface, and also to load species of interest inside the pores. Adequate control of the synthesis conditions and functionalization of the mesoporous surface are critical factors in the design of new systems that are suitable for use in specific medical applications. Simultaneously, the use of appropriate characterization techniques in the several stages of design and manufacture of mesoporous particles allows us to ascertain the textural, structural and compositional modifications induced during the synthesis, functionalization and post-in vitro assays processes. In this scenario, the present paper shows, through several examples, the role of transmission electron microscopy and associated spectroscopic techniques in the search for useful information in the early design stages of mesoporous systems, with application in the fields of tissue regeneration and drug delivery systems.

## 1. Introduction

Research on mesoporous materials is booming, with considerable effort devoted to a wide variety of exciting applications for the well-being of society. A whole range of materials has emerged from the discovery of these mesoporous solids, from catalytic to medical [1] and nanotechnological [2] applications. Chemistry in confined spaces, which became especially important in the 1970s due to technology based on zeolites, was the basis for a great deal of experimental work. In 1972, Mobil Corporation developed a procedure to transform methanol into gasoline using Zeolite Socony Mobil (ZSM-5) as a catalyst [3]. During this period, research was focused onto discover materials with large pores, and the first mesoporous silica material was produced. These porous silica solids were obtained by combining the inorganic silica component with amphiphilic surfactant molecules [4,5]. Ordered mesoporous ceramics were also synthesized in the early nineties, when Mobil Oil Corporation researchers [6,7] produced materials with larger pores than zeolites to improve their applicability as adsorbents, catalysts and catalysts scaffolds. 

Subsequently, the search for new mesoporous solids has increased considerably in the 21st century and new materials have arisen through the development of new synthesis pathways, as is the case of mesoporous solids. Tremendous experimental efforts have led to improving the important and complex catalytic potential of these materials [8], and have also been responsible for new developments that have established the field of mesoporous materials as an active and important part of material research for the future. Complex techniques for structural characterization and elucidation of the formation mechanism, new developments in metal oxide-based synthesis, and applications in medicine, nanomedicine, magnetism, photo catalysis, fuel cells, thermo-electrics and nano-electronics, are some of the areas of research where mesoporous materials are having an important impact [9]. From a technological point of view, besides their catalytic applications, the pores and cages of such materials are being tailored for potential use in both the electronics and pharmaceutical industries, which will have an enormous impact on the welfare of society.

In the health field, the work of Professor Vallet-Regí brought to light the contribution of mesoporous materials to the world of medical research. Her pioneering work was pointed toward bone tissue regeneration. Furthermore, at the beginning of this century, she proposed, for the first time, the use of mesoporous materials as drug delivery systems [10]. Since then, silica-based ordered mesoporous materials have received growing interest from the biomaterials scientific community due to their ability to host different guest molecules [11]. The parameters that govern drug adsorption and release processes mainly depend on the textural and structural properties of the host–matrix. In the last few years, this research has been expanded to the design of functionalized mesoporous materials with the ability to release bioactive molecules, hence improving our knowledge of the fundamental parameters driving the behavior of these materials [12,13,14]. This scientific effort and the methodology developed have become the cornerstone for an in-depth understanding of the drug delivery systems that are so popular in the field today. The complexity of these hybrid systems increases when trying to adapt their properties to specific clinical needs. In many cases, it is necessary to organically modify the silica walls by covalent bonding of functional groups. This functionalization process results in hybrid materials that can act as host matrices for a wide range of drugs through weak interactions. 

With the remarkable advances in the preparation of the mesoporous materials developed by Professor Vallet-Regí and co-workers, new horizons are opening up in the development of new organic–inorganic hybrids in the field of biology. This necessarily facilitates precise control over the processes of therapeutic substances, and guarantees the possibility of increasing the efficacy of the therapy. The targeting ability of these new nanodevices will make it possible to establish tailored dosing routines, with a significant reduction in the side effects associated with some pathologies, such as cancer; it should also lead to a more appropriate distribution of medical resources. In many cases, silica mesoporous nanoparticles are being used, even though the origin of these ordered mesoporous ceramics is mainly from the catalysis industry. Mesoporous materials, due to this pioneering research, have found very promising applications in the medical world, which promotes their drug delivery capabilities and tissue engineering potential [15,16,17,18,19,20,21,22,23]. 

The meso-scale order is very sensitive to both the synthesis pathway and annealing time, with various local fluctuations in the former causing local structural variations. Mesoporous materials exhibit poor crystallinity and inevitably contain various kinds of defects. Powder X-ray diffraction (XRD) profiles of mesoporous silica crystals show, in most cases, a few maxima so that structural resolution by powder XRD alone is not sufficient. Transmission electron microscopy (TEM) can overcome these limitations and has strong potential for structural studies of nano-crystals. It is well known that electrons, with a shorter wavelength than X-rays, interact more strongly with condensed matter, allowing us to collect the same structural information in a crystal about 7–8 orders of magnitude smaller. Complementary compositional information can be obtained through associated spectroscopic techniques such as X-ray energy dispersive (EDS) and electron energy loss (EELS) spectroscopies [24]. The aim of this paper is to review how the ensemble of transmission electron microscopy and associated EDS and EELS tools have been very useful to elucidate details on the textural, structural, microstructural and compositional properties of mesoporous materials for biomedical applications in the fields of tissue regeneration and drug delivery in collaboration with Professor Vallet-Regí.

In order to get a proper insight into the biological properties of these systems, we need access to two very important features of the mesoporous silica nanoparticles: the pore diameter and their distribution. Two TEM images with incidences parallel and perpendicular to the channel can provide conclusive evidence of the P6mm symmetry as a 2D hexagonal structure and the one-dimensional channel system characteristic of mesoporous materials. Electron microscopy observations combined with powder XRD experiments have been used to solve the structure of MCM-41 [6]. This exhaustive characterization, by combining HRTEM and electron crystallography, is the key to establishing the relationship between structure and properties, opening the way to outstanding new applications. The phase information for structural factors contained in the Fourier patterns obtained from electron microscopy images is essential to identify the structure.

## 2. Materials and Methods

The mesoporous materials studied in this work were synthesized by the sol–gel method as previously reported in the literature [7,25,26,27]. The mesoporosity of the silica-based materials was checked by X-ray diffraction (XRD) and surface area studies. The XRD study was performed in a Philips X´Pert MPD (Cu kα radiation) (Panalytical, Malvern Panalytical DV, Almelo, The Netherlands) diffractometer in the 2θ range of 1–10°. Surface area was obtained by N_2_ absorption measurements in a porosimeter (Micromeritics ASAP 2010, Micromeritics, Norcross, GA, USA). The pore size was calculated according to the Barret–Joyner–Halenda and Kruk–Jaroniec–Sayari approach [28,29,30]. Once the average characterization of the mesoporous materials has been done with the above techniques, further characterization through transmission electron microscopy (TEM) provides direct observation of the particle size, morphology, microstructural details as well as compositional information from the associated spectroscopies. There are two main imaging techniques: conventional transmission electron microscopy (CTEM) and scanning transmission electron microscopy (STEM). In CTEM, a parallel beam illuminates a broad area of the sample, the beam is transmitted and the objective lens, depending on its current, obtains a diffraction pattern or an image of the sample that delivers important crystallographic information. In the STEM mode, a convergent beam is focused on a small area of the sample and then scanned over the surface; at each scanning position the beam is transmitted. The use of different detectors provides different data. Perhaps the most extended mode is the high angle annular dark field (HAADF) in which the electrons diffracted at a high angle are collected, providing a contrast in the image that is highly dependent on the atomic number, Z. In this way, the images render compositional information when heavy atoms with different atomic numbers are present. Moreover, in the STEM-HAADF mode, the use of a highly focused beam is very advantageous since it allows the simultaneous acquisition, with the corresponding STEM-HAADF image, of EELS or EDS spectra. If the beam is small enough to be positioned on a single atomic column, this information is gathered with atomic resolution. For this purpose, probe-corrected aberration microscopes are required. In this work, both CTEM and STEM modes have been used coupled with EDS and EELS spectroscopies. Transmission electron microscopy images, selected area electron diffraction (SAED) and medium resolution scanning transmission electron microscopy (STEM) experiments were carried out on a JEOL JEM300FEG electron microscope equipped with an ISIS 300 X-Ray microanalysis system (Oxford Instruments, Scotts Valley, CA, USA) with a LINK “Pentafet” EDS detector (Oxford Instruments, Scotts Valley, CA, USA). The atomic resolution study was carried out on a JEOL JEMARM200cF aberration-corrected STEM electron microscope (cold emission gun) (JEOL Ltd., Tokyo, Japan) operating at 200 kV (probe size ~0.08 nm) and electron energy loss spectroscopy (EELS) experiments were conducted using a Gatan-Enfina Spectrometer (Gatan Inc., Pleasanton, CA, USA). The samples described in this manuscript were ultrasonically dispersed in n-butanol and transferred to coated copper or nickel grids for transmission electron microscopy (TEM) characterization. In addition, further information concerning the drug loading, functionalization and compositional modifications was supported by Fourier-transformed infrared (FTIR, Oxford Instruments, Scotts Valley, CA, USA) spectroscopy and X-ray fluorescence (Panalytical, Malvern Panalytical DV, Almelo, The Netherlands).

## 3. Results

Ordered mesoporous ceramics are unique materials characterized by an ordered mesostructure of pores and disordered arrangement at the atomic level. By using synthetic templates, the obtained ceramics presented unique structural properties, such as a stable and ordered mesoporous structure, high surface area, large pore volume, regular and tunable mesopores size (2–50 nm) and homogeneous pore morphology. The channels, cages or pores formed within the materials and supported/separated by an amorphous silica wall, are arranged periodically on a two-dimensional lattice, resembling the arrangement of atoms or molecules in ordinary crystals. Thus, the materials might be thought of as “cavity-crystal” [31]. This ideal situation can be modified by varying the experimental parameters either at the bulk or nanometer scale. Two examples indicating how TEM can show these changes are described as follows.

**Figure 1:**
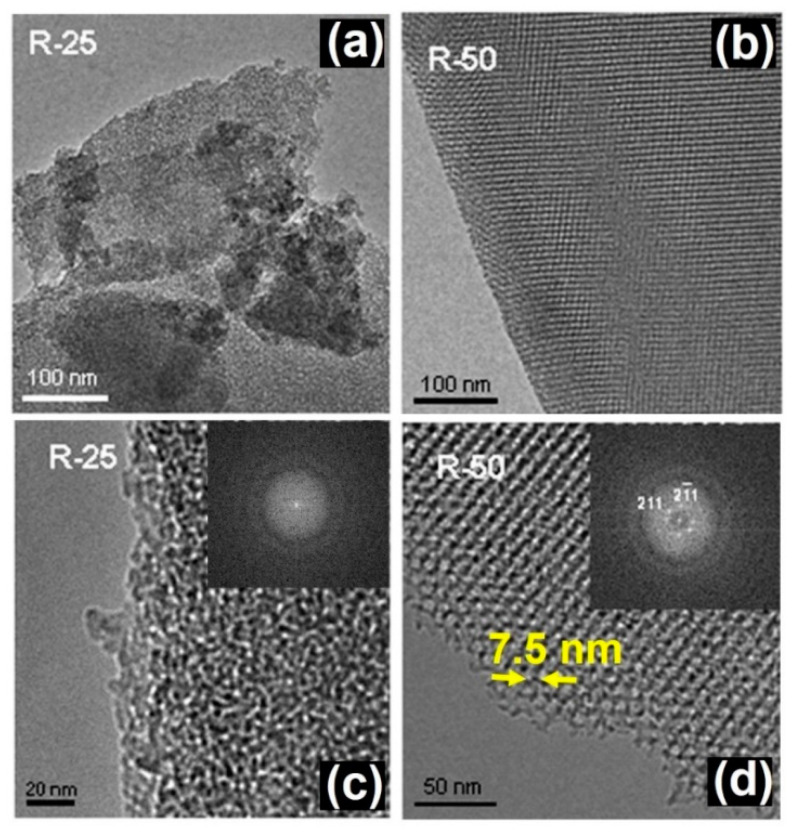
TEM images of RT samples with different surfactant/silica ratio corresponding to R25 (**a**,**c**) and R50 (**b**,**d**). Adapted with permission from [32]. Copyright 2008 Trans Tech Publications Ltd. (Stafa-Zurich, Switzerland).

In this context, it has been shown how the macroscopic shape and mesoporosity of SiO_2_-based mesoporous microparticles can be modified using the evaporation-induced self-assembly method through two different routes: room temperature (RT) and aerosol-assisted synthesis (A-A) using different surfactant/silica precursor ratios [32]. According to small-angle XRD, the RT samples exhibited a cubic symmetry (Ia-3d) while the A-A series could be assigned to a 2D hexagonal (P6 mm) structure. The TEM study of the RT samples showed an irregular particle morphology evidencing clear differences in the particle size and microstructural features of samples depending on the surfactant/silica ratio. Differences in the particle size were observed in the typical low magnification images of samples (Figure 1a,b) with a surfactant/silica ratio of 25 (R25) and 50 (R50), respectively. Moreover, a very poorly ordered mesoporous arrangement was observed in sample R25 (Figure 1c) while R50 showed an ordered contrast distribution, with periodicities of 7.5 nm (Figure 1d).

**Figure 2:**
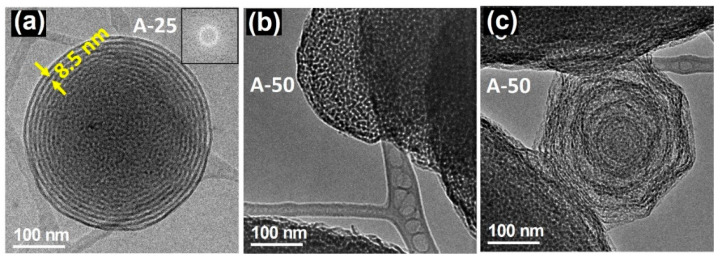
TEM images of A-A samples with different surfactant/silica ratio corresponding to (**a**) R25 and (**b**,**c**) R50. Adapted with permission from [32]. Copyright 2008 Trans Tech Publications Ltd. (Stafa-Zurich, Switzerland).

In contrast to the RT samples, the A-A particles exhibited a spherical morphology as observed in Figure 2a. When the surfactant/silica ratio was modified, differences in the microstructure were again found. For A25, two different microstructures coexist in the inner and outside parts. The interior is associated with the original mesoporous structure, which clearly shows a very poor crystalline arrangement. The hexagonal porous arrangement expected from the XRD data, shown in Figure 2 of reference [32], was not visualized, probably due to a partial decomposition under the electron beam. A lamellar arrangement appears in the outer part with a periodicity of 8.5 nm between layers. In the case of A50, two kinds of particles were found, spherical ones (Figure 2b) and others, in a clear process of decomposition, which exhibited a polygonal shape (Figure 2c). This situation derives from the collapse of the lamellar structure after heat treatment, resulting in a poorly ordered arrangement.

**Figure 3:**
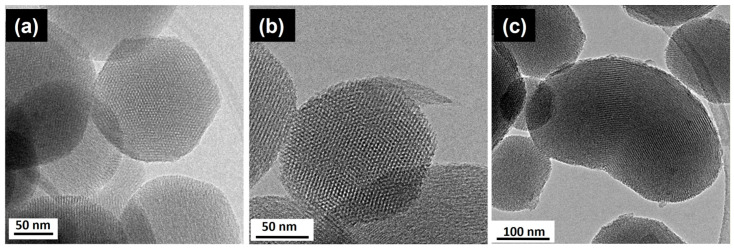
HRTEM images from (**a**) MSN and amine-functionalized MSN with TEOS:APTES mole ratio (**b**) 90:10—MSN-10N and (**c**) 70:30—MSN-30N. Adapted with permission from [43]. Copyright 2018 Elsevier (Amsterdam, The Netherlands).

One of the great advantages of these ordered mesoporous materials is the great versatility of the synthetic process, which allows production in bulk, but also as microcapsules and even as nanoparticles (MSNs). For these purposes, MSNs require the appropriate functionalization of their surface with targeting agents [33,34], drugs [35,36,37,38], and stimuli-responsive molecular gates [39]. Specifically, organosilanes containing a reactive functional group such as amino-organosilanes are efficient linkers for the covalent functionalization of MSNs [40,41,42]. SiO_2_ MSNs were prepared [43] with different amounts of aminopropyltriethoxysilane (APTES) by the condensation method. A careful characterization revealed that the surface charge and nanoparticles’ morphology were strongly influenced by the amount of APTES. In particular, the TEM study (see Figure 3) evidenced the stabilization of single crystal nanoparticles with a highly-ordered hexagonal mesoporous structure. It should be noticed that morphological differences were observed when the TEOS/APTES ratio was modified. In fact, particles with a TEOS/APTES ratio of 90:10 (MSM-10N) usually exhibit a similar hexagonal polyhedral morphology and a mesoporous structure (see Figure 3a,b). However, for a higher APTES concentration (TEOS/APTES ratio 70:30), the resulting MSN-30N particles showed curved shapes (bean-like morphology) (Figure 3c). The HRTEM characterization confirmed the hexagonal structure (P6mm) characteristic of MCM-41, as observed in the hexagonal array or mesopores aligned parallel to the morphological long axis in the three figures.

**Figure 4:**
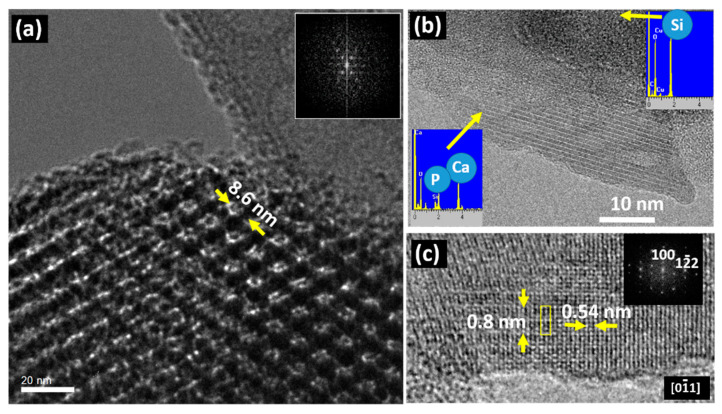
(**a**) TEM image of SBA-15 before immersion for 60 days in SBF. The corresponding Fast Fourier Transform (FFT) is shown in the inset; (**b**) TEM image of an acicular particle formed after 60 days in SBF. EDS spectra performed on the particle and the amorphous matrix are shown as insets; (**c**) HRTEM detail of an acicular particle and its FFT. Adapted with permission from [45]. Copyright 2005 Elsevier (Amsterdam, The Netherlands).

In addition to the information concerning the control of the synthesis procedures, as shown in the above two examples, TEM can also provide useful information regarding the bioactivity of these mesoporous systems in relation to their bioactivity. The following examples illustrate this ability. In the field of bone implants and prostheses, bioactivity is a property that involves the ability of a substance to form interfacial bonds with osseous tissues when in contact with a physiological fluid, which always involves the formation of a hydroxy-carbonate apatite layer. Although the mechanisms of apatite nucleation and crystallization are not fully understood, the characteristics of both substrates and fluids seem to have an appreciable influence. As far as the solution is concerned, parameters such as pH, temperature and ionic concentration determine the type of calcium phosphate formed as well as its precipitation rate. On the other hand, the presence of silanol groups and porosity seems to be crucial in the formation of the apatite layer. In this regard, mesoporous silica materials, with a pore size ranging from 2 to 50 nm and surface silanol and siloxane reactive groups, are promising candidates as bioactive materials. Taking into account these ideas, Professor Vallet-Regí studied the bioactivity properties of three mesoporous materials with different structural and textural properties, SBA-15, MCM-48 and MCM-41, by means of in vitro assays in simulated body fluid (SBF) [44], which has roughly the same ionic concentration of human plasma, at 37 °C. The objective of these assays was to evaluate the influence of the distinct structural and textural features of the three selected mesoporous in contact with the SBF. A complete study, using several techniques was performed [45], and suggested a modification in the SBA-15 and MCM-48 surfaces since they were covered with carbonate hydroxyapatite [45,46], although with different kinetics. This took place in 30 days for SBA-15 while double this time was required for MCM-48. The MCM-41 was not modified, even after longer periods of time. Pre-treatment HREM images showed the pore size and hexagonal pore distribution of the raw material, as observed in Figure 4a for the SBA-15. After the indicated period in SBF, the apatite formation was confirmed on the SBA-15 surface since acicular particles (Figure 4b) appeared over the mesoporous surface, which was simultaneously degraded.

The crystalline nature of the acicular particles can be observed in Figure 4b, which were placed in an amorphous matrix, containing only Si, which comes from the original mesoporous matrix that deteriorated after the SBF treatment. In the acicular particle, the EDS analysis indicates the presence of Ca and P, which are characteristic of the apatite. The measured periodicity as well as the FFT agree with the formation of hydroxyapatite on the surface of the mesoporous matrix (Figure 4c). In fact, compositional differences between the crystalline and amorphous areas were detected; the crystalline region comprises Ca and P while the amorphous one contains Si as expected from the starting mesoporous matrix. A similar situation has been found for MCM-48, but it was different for MCM-41, since it did not exhibit any change on its surface, even after two months in contact with SBF. This fact could be understood by considering that MCM-41 shows a significantly lower concentration of silanol groups in comparison with SBA15 and MCM-48, which hinders the nucleation process of the apatite [47,48,49]. Furthermore, the textural and structural properties can also influence apatite nucleation, which is easier in more opened frameworks with accessible channels [50,51,52]. The mesoporous system that best fulfils these requirements is SBA15 because it exhibits the largest pore size (8.8 nm) and an interconnected 3D framework of pores. On the other hand, a lower pore size of 3.6 nm is found in MCM-48 and MCM-41. In spite of the similarity in pore size, the worst kinetics is found for MCM-41 due to its less accessible one-dimensional arrangement of pores, as well as to the lower concentration of silanol groups covering the pore surfaces. 

These studies indicated that mesoporous silica matrices are good candidates for bone regeneration in a period of more than a month, as a consequence of their bioactive character, being able, at the same time, to act as a host for different drugs (antibiotics, anti-inflammatories, anti-carcinogens), but also for other biologically active substances to accelerate the process of formation of new bone (peptides or growth factors), to achieve a controlled release of both types of molecules in the first few days following the implantation. 

**Figure 5:**
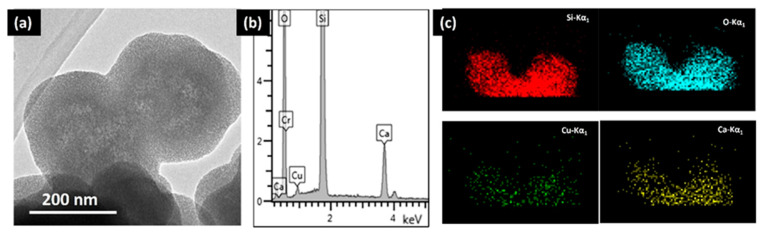
(**a**) TEM image of Cu-MBG 2%; (**b**) EDS spectrum of Cu-MBG 2%; and (**c**) EDS mapping of Cu_MBG 2. Adapted with permission from [53]. Copyright 2017 Elsevier (Amsterdam, The Netherlands).

When dealing with the synthesis of hybrid mesoporous materials of higher compositional complexity, TEM and its associated spectroscopies can provide valuable information. For instance, mesoporous bioactive glasses (MBGs) constitute another exciting system for bone-tissue regeneration. These systems combine the texture of mesoporous matrices with the properties of bioactive sol–gel glasses [46,53,54,55,56,57,58,59,60]. The higher bioactivity of MBGs compared to conventional bioactive glasses, makes them into ideal ingredients for bone regeneration [53]. Furthermore, additional biological functions such as the anti-bacterial activity, stimulation of osteogenesis and angiogenesis can be improved by including controlled amounts of metallic ions with therapeutic effect [60,61]. For instance, Cu ions exhibit recognized therapeutic properties. In this context, the framework of a binary SiO_2_-CaO system was modified with different amounts of copper ions through an ultra-sound-assisted base catalyzed sol–gel method. EDS analysis confirmed the incorporation of Cu inside the lattice (Figure 5) for a MBG sample containing a 2% molar percentage of Cu (Cu_MBG 2%). Electron micrographs showed that the nanoparticles contain mesopores throughout their internal structure, in the form of a worm-like system (Figure 5a). Quantitative analysis using STEM-EDS mapping showed that Cu and Ca ions were evenly distributed within the particle (Figure 5b,c). The antibacterial activity against different human pathogenic bacteria was tested, which demonstrated that Cu_MGB 2% inhibited the bacterial growth and was also able to counteract the formation of biofilm produced by Staphylococcus epidermidis, and even to favor its dispersion.

**Figure 6:**
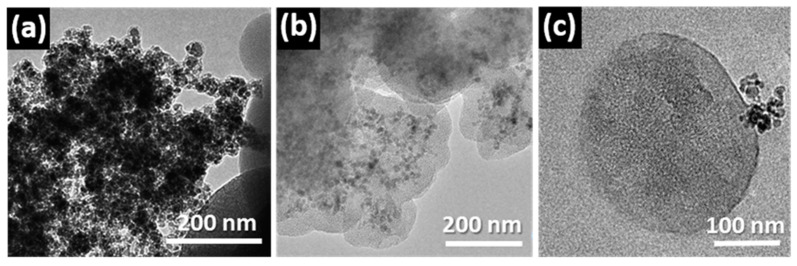
Low magnification TEM images of the mesoporous particles after attempting the encapsulation of (**a**) γ-Fe_2_O_3_ without coating; (**b**) γ-Fe_2_O_3_-1 coated with APTEs and (**c**) γ-Fe_2_O_3_-2 coated with silica. Adapted with permission from [73]. Copyright 2012 Royal Society of Chemistry (London, UK).

In a similar way, the outstanding textural properties of the mesoporous materials can also be tuned for the design of mesoporous magnetic nanocomposites (MMNs) [11,62], which exhibit enormous potential for biomedical and biotechnological applications such as drug/gene delivery systems [63,64,65], NMR imaging [62], enzyme immobilization [66], cellular uptake [67], and as thermoseeds for cancer treatment by hyperthermia [68,69]. These systems incorporate magnetic nanoparticles inside the mesoporous material, combining their outstanding properties. Different strategies for synthesis have been developed to embed encapsulated nanoparticles into the mesoporous silica matrices [62,70,71]. In these processes, the appropriate surface functionalization is a critical factor for the incorporation of the magnetic nanoparticle. In this context, the preparation of mesoporous silica microspheres based on a sol–gel process in the presence of a cationic structure directing agent [72] combined with three different ferrofluids: (a) pure γ-Fe_2_O_3_ without coating; (b) γ-Fe_2_O_3_-1 coated with APTEs and (c) γ-Fe_2_O_3_-2 coated with silica (Figure 6) was tested in order to obtain MMNs [73]. TEM studies helped to evaluate the effectiveness of the synthetic method. Figure 6c shows TEM images obtained from the three attempts indicating that only γ-Fe_2_O_3_-1 nanoparticles were successfully incorporated into the SiO_2_ mesoporous particles. 

**Figure 7:**
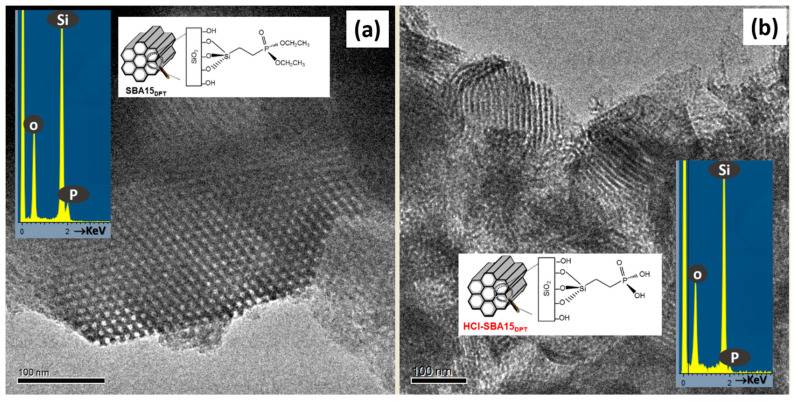
HRTEM images corresponding to (**a**) SBA15 DPT and (**b**) HCl-SBA15DPT. The images show a schematic representation of the functionalization and the typical EDS analysis. Adapted with permission from [83]. Copyright 2012 Royal Society of Chemistry (London, UK).

HRTEM and related techniques can also be key tools for unveiling the structural and compositional details of mesoporous systems for drug delivery applications. Drug delivery involves a complex process [24,74,75] that can be explained through a series of steps involving penetration and drug dissolution in the release medium and drug diffusion through the channels. The surface chemical nature and the structural features of the mesoporous materials govern the release of molecules and determine the drug delivery profiles [36,76,77,78]. Pore diameter, connectivity and structure are critical factors that influence the drug release kinetics [10,79,80,81]. On the other hand, the functionalization of mesoporous matrices is fundamental to the performance of these materials [24,36,75,81,82] since the silica surface is modified by grafting organic groups selective to the chemical nature of the drug to be hosted, thus increasing the host–guest interaction. In some cases, functionalization requires post-synthesis treatments involving harsh conditions that can affect the mesostructural order [83]. In this scenario, HRTEM is a useful tool to control whether any modification takes place. For instance, SBA-15 mesoporous material bearing ≡Si(CH_2_)_2_P(O)(OCH_2_CH_3_)_2_ (SBA15 DPT) was synthesized and then treated with 35 wt% HCL (HCl-SBA15DPT) [24] to convert ethyl-phosphonate groups to ethylphosphonic acid groups. HRTEM revealed that the acid-treatment induced a noticeable loss of mesostructural order, with only small crystalline domains remaining, as observed in Figure 7. 

The EDS analyses (see insets in Figure 7) of both samples indicated the presence of P in accordance with the external functionalization. The in vitro delivery tests indicated that the structural damage did not influence cargo loading but it severely affected the release of molecules confined within the mesopores. 

**Figure 8:**
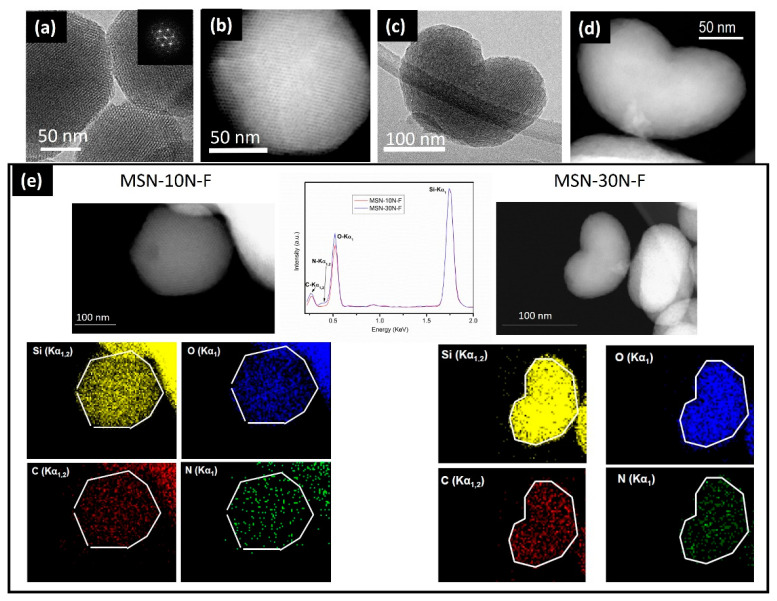
(**a**,**b**) Characteristic HRTEM and STEM images corresponding to the MSN-10N-F sample; (**c**,**d**) characteristic HRTEM and STEM images corresponding to the MSN-30N-F sample; (**e**) EDS spectra of MSN-10N-F (left image) and MSN-30N-F (right image) areas and corresponding Si, O, N and C elemental maps (bottom part). Adapted with permission from [43]. Copyright 2018 Elsevier (Amsterdam, The Netherlands).

Amino-organosilanes MSNs [43], such as MSN-10N and MSN-30N described above, exhibit an outer surface that can covalently attach active targeting agents such as folate groups (F). An HRTEM-STEM-EDS study was performed in order to evaluate the effect of the grafting with folic acid. The HRTEM and STEM images of the MSN-10N-F indicate a mesoporous hexagonal structure (Figure 8a,b) similar to those before grafting (see Figure 3). When the APTES ratio increases (MSN-30N-F), TEM images suggest a loss of ordering (Figure 8c,d) and show defective areas. Figure 8e shows the EDS spectra corresponding to the previous images of MSN-10N-F and MSN-30N-F and the collected Si, O, N and C elemental maps. These spectra show the same distribution for Si in both samples. Nevertheless, O and N contents seem to be subtly higher in MSN-30N-F. Additionally, a homogeneous distribution of N and C can be observed in the elemental maps confirming the homogeneous F coating for both samples. These co-functionalized MSNs, MSN-10N-F and MSN-30N-F were tested as selective targeted nanoparticles. In the case of MSN-10N-F, a cooperative effect between aminopropylsilane and folic acid (FA) was observed, increasing the MSMs internalization in tumoral cells, which opens up very interesting possibilities for the design of more effective targeted nanovehicles.

**Figure 9:**
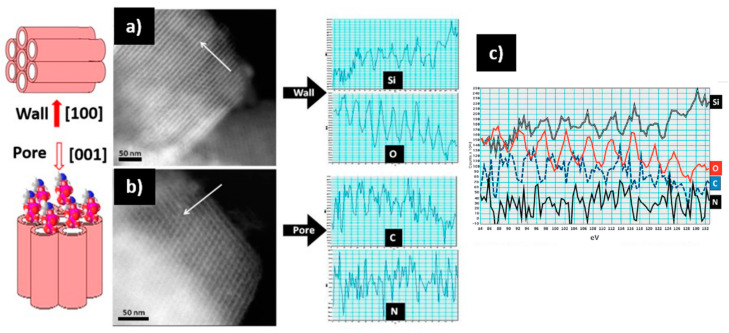
STEM-HAADF images of SBA-15 mesoporous matrix loaded with zolendronate molecules along the wall [100], and the pore [001] and the corresponding EELS intensity profiles (see arrows): (**a**) along [100] the EELS profile shows the presence of Si and O; (**b**) along [001] the pores are observed and the profile reveals the presence of N and C inside; (**c**) along the same direction [001], showing the Si, O, C, and N profiles [84]. Adapted with permission from [84]. Copyright 2010 Royal Society of Chemistry (London, UK).

Electron microscopy can go further in the characterization of these systems, proving the presence of drugs in the inner part of the functionalized mesopores by using the STEM-EELS capabilities and employing a spherical aberration (Cs)-corrected microscope. The aberration correction in the STEM mode renders very small probes that provide high sensitivity and high spatial resolution, which offers outstanding analytical capabilities in combination with EELS and EDS spectroscopic techniques. Using this instrumentation, Vallet-Regí and co-workers [84] were able to demonstrate, for the first time, the detection of zolendronate molecules inside the pore channels of an SBA-15 mesoporous matrix. This can be observed in Figure 9, where STEM-HAADF images of this system along the [100] and [001] directions are displayed with the corresponding EELS intensity profiles, evidencing the presence of Si and O across the wall (see Figure 9a) and C and N, which are characteristic of the zolendronate molecules, along the pore direction (see Figure 9b). Along this profile, the Si and O signal appear at the same position (white contrast) alternating with C and N (dark contrast), proving that zolendronate is inside the pore but not in the mesoporous wall (see Figure 9c).

Since the beginning of the research on mesoporous silica-based materials within the biomedical field up to now, the complexity of these systems has grown enormously. The first nanotechnological application was the controlled release of molecules loaded in the mesopores of MCM-41 [10]. From this discovery, molecular gates, based on intelligent systems and external stimuli, that avoid the premature release of the drug, were designed. These systems involve modifying the mesoporous surface by anchoring different types of compounds such as magnetic nanoparticles, specific molecules, proteins or polymers [85,86,87] able to respond to some external stimuli. The treatment of complex pathologies, such as cancer, requires multidrug release by hybrid mesoporous silica nanoparticles [15]. Furthermore, hybrid enzyme-polymeric capsules/mesoporous silica nanodevices have been designed in order to support the penetration of the nanomedicines. Similarly, complex systems are also being studied for the treatment of osteoporosis and bone infections. 

In order to understand the behavior of these complex/hybrid mesoporous materials, it is crucial to successfully acquire direct images with the best possible resolution and in a non-destructive fashion, to identify the chemical composition, locate dopants, and verify the organic functionalization. Unfortunately, besides the high sensitivity of the mesoporous matrices, the organic functionalization is easily destroyed under the electron beam. Lowering the acceleration voltage [88] reduces the knock-on damage effects but increases the radiolysis [89] effects, leading to critical damage in biological and organic systems. Nevertheless, when the voltage is reduced, the inelastic scattering increases and the contrast is also increased, which is sought after for biological and organic sample imaging. In addition, the spectroscopy efficiency is also improved and the delocalization effect decreases, easing the imaging interpretation. The introduction of aberration correction allows working at low energies, which keeps the atomic resolution. At low voltage, the major limitation to resolution arises from the chromatic aberration. This can be improved by the incorporation of a monochromator in the source. In addition, new developments in monochromators have made possible vibration and valence loss EELS [90]. This is a promising non-destructive method to analyze the organic functionalized surface of hybrid materials. On the other hand, the use of a low electron dose usually renders a low signal-to-noise ratio in images acquired with conventional charge-coupled device cameras, but the implementation of direct-detection cameras [91] provides an efficient solution. For all of these reasons, studying hybrid mesoporous materials by adjusting both the voltage and electron dose in the microscope is mandatory in order to get the best results, which will always involve reaching a compromise between resolution, contrast and damage. Using the latest technological advances such as aberration TEM, monochromator and efficient detector systems can highly improve the image quality. In addition, radiolysis damage can be reduced by using a specimen holder cooled by liquid nitrogen or liquid helium [92].

## Data Availability

Not applicable.
